# Genetics in Idiopathic Pulmonary Fibrosis Pathogenesis, Prognosis, and Treatment

**DOI:** 10.3389/fmed.2017.00154

**Published:** 2017-09-25

**Authors:** Amarpreet Kaur, Susan K. Mathai, David A. Schwartz

**Affiliations:** ^1^Department of Medicine, University of Colorado Denver School of Medicine, Aurora, CO, United States; ^2^Department of Medicine, Division of Pulmonary Sciences and Critical Care Medicine, University of Colorado Denver School of Medicine, Aurora, CO, United States

**Keywords:** idiopathic pulmonary fibrosis, MUC5B, pulmonary fibrosis, interstitial lung disease, telomeres

## Abstract

Idiopathic pulmonary fibrosis (IPF), the most common form of idiopathic interstitial pneumonia (IIP), is characterized by irreversible scarring of the lung parenchyma and progressive decline in lung function leading to eventual respiratory failure. The prognosis of IPF is poor with a median survival of 3–5 years after diagnosis and no curative medical therapies. Although the pathogenesis of IPF is not well understood, there is a growing body of evidence that genetic factors contribute to disease risk. Recent studies have identified common and rare genetic variants associated with both sporadic and familial forms of pulmonary fibrosis, with at least one-third of the risk for developing fibrotic IIP explained by common genetic variants. The IPF-associated genetic loci discovered to date are implicated in diverse biological processes, including alveolar stability, host defense, cell–cell barrier function, and cell senescence. In addition, some common variants have also been associated with distinct clinical phenotypes. Better understanding of how genetic variation plays a role in disease risk and phenotype could identify potential therapeutic targets and inform clinical decision-making. In addition, clinical studies should be designed controlling for the genetic backgrounds of subjects, since clinical outcomes and therapeutic responses may differ by genotype. Further understanding of these differences will allow the development of personalized approaches to the IPF management.

## Introduction

Idiopathic pulmonary fibrosis (IPF) is the most common of the idiopathic interstitial pneumonias (IIPs). IPF is characterized by progressive scarring of the lung parenchyma, which leads to dyspnea and declining pulmonary function and eventually to respiratory failure. The median survival after diagnosis of IPF is 3–5 years (1). In 2011, the American Thoracic Society/European Respiratory Society issued a new classification scheme in which they defined IPF as a specific form of chronic, progressive fibrosing interstitial pneumonia of unknown etiology, occurring mainly in older adults and associated with radiological and/or histopathological pattern of usual interstitial pneumonia (UIP) (2). The prognosis of IPF remains poor despite recently approved medical therapies (3, 4).

Numerous epidemiologic and genetic studies illustrate that genetic and environmental factors contribute to the risk of IPF (5, 6). The most convincing early evidence to support a genetic basis for IPF came from twin studies and studies focusing on familial clustering of the disease, a syndrome termed familial interstitial pneumonia (FIP) (7–9). Recent studies have identified several specific genetic variants that confer risk for development of IPF (10, 11). Discovery of disease-associated genetic variants has improved our understanding of the ways inherited risk factors influence disease risk. However, fundamental questions persist regarding the ways in which complex genetic risk factors interact with environmental exposures to influence disease pathogenesis.

In this review, we briefly discuss the current literature regarding the role of common and rare variants in disease pathogenesis and prognosis and how this may influence clinical management in the future. Genetic variants and loci associated with IPF involve abnormalities in alveolar stability, host defense, cell–cell barrier function, and cell senescence, all of which are all thought to contribute to the pathogenesis of IPF. We conclude by discussing how treatment decisions might be affected by these findings and how better understanding of genetic variation and disease could allow for a more personalized approach to the treatment of IPF.

### Rare and Common Variants Associated with IPF

Genetic variants, both rare and common, are associated with sporadic and familial forms of pulmonary fibrosis. Numerous rare variants (those with minor allele frequency of <0.1%) play a role in FIP (≥2 members of the same family with interstitial pneumonia; FIP) (Table 1). Familial studies have identified FIP-associated variants related to alveolar stability [*SFTPC* (12, 13), *SFTPA1* (14), *SFTPA2* (15), ATP-binding cassette-type 3 (*ABCA3*) (16), and *NAF1* (17)] as well as five genes linked to telomere biology [*TERT* (18), *TERC* (18), *DKC1* (19), *TINF2* (7, 20), *RTEL1* (21–23), and PARN] (24).

**Table 1:** Rare variants in idiopathic pulmonary fibrosis.

Gene	Gene function	Pathological consequence of mutation	Reference
*SFTPC*	Component of surfactant fluid	Altered trafficking and disrupted proteostasis, increased endoplasmic reticulum (ER) stress	(25–27)
*SFTPA2*	To modulate innate and adaptive immunity	Increase in ER stress	(15, 25, 28)
*ABCA3*	Transport of lipids across plasma membrane	Retention of lipids in the ER, ER stress, and apoptotic signaling	(29–31)
*TERT*	Enzyme in telomerase complex	Telomere shortening	(7, 18, 27, 32–36)
*TERC*	Template in telomerase complex	Telomere shortening	(7, 18, 27, 32–37)
*DKC1*	Stabilization of the template in telomerase complex	Telomere shortening	(19, 27, 38)
*TINF2*	Telomere maintenance	Telomere shortening	(20, 39)
*RTELI*	DNA helicase	Telomere shortening	(21, 22, 40)
*PARN*	mRNA stability	Telomere shortening	(21, 24)

Common variants (defined as minor allele frequency of >5%) also appear to play a role in FIP risk (1). The most widely replicated risk variant (rs35705950), located in the promoter region of *MUC5B*, was initially identified in a combined linkage and association study (41) and has been strongly associated with IPF and FIP. Two large GWAS of IPF subjects (both familial and sporadic) with controls have been conducted in pulmonary fibrosis (10, 11). In addition to confirming the role of *TERT* at 5p15, *MUC5B* at 11p15, and the 3q26 region near *TERC*, the GWAS identified seven newly associated loci, including *FAM13A* (4q22), *DSP* (6q24), *OBFC1* (10q24), *ATP11A* (13q34), *DPP9* (19q13), and chromosomal regions 7q22 and 15q14-15 among others that have been nominally associated (Table 2).

**Table 2:** Common variants in idiopathic pulmonary fibrosis (IPF).

Risk allele(s)	Gene	Gene function	Observed effect of risk variant on survival in IPF	Reference
rs408392	*IL1RN*	Inhibitor of pro-inflammatory effect of IL-1alpha and IL-1beta		(27, 42)
rs419598				
rs2637988				
rs4073	*IL8*	Pro-inflammatory cytokine	Reduced	(43, 44)
rs2227307				
rs2609255	*FAM13A*	Signal transduction		(10)
rs3775291	*TLR3*	Pathogen recognition and activation of innate immunity	Reduced	(45)
rs2736100	*TERT*	Enzyme in telomerase complex maintaining telomere length	Reduced	(10, 27, 46, 47)
rs2395655	*HLA-DRB1*	Major histocompatibility complex—immune system		(48)
rs2076295	*DSP*	Tightly links adjacent cells		(10)
rs11191865	*OBFC1*	Stimulates the activity of DNA polymerase-alpha-primase		(10)
rs35705950	*MUC5B*	Influence on rheological properties of airway mucus, mucociliary transport, and airway defense	Improved	(10, 11, 27, 41, 49, 50)
rs7934606	*MUC2*	Mucin production		(10)
rs111521887	*TOLLIP*	Regulator of innate immune responses mediated by toll-like receptor and the transforming growth factor β signaling pathway	Reduced	(11)
rs5743894				
rs2743890				
rs1278769	*ATP11A*	Phospholipid translocation		(10)
rs7144383	*MDGA2*	Cell–cell interaction		(11)
rs1981997	*MAPT*	Promotes microtubule assembly and stability		(10)
rs17690703	*SPPL2C*	Protein cleavage		(11)
rs12610495	*DPP9*	Cell–cell adhesion		(10)
rs1800470	*TGFB1*	Set of peptides that controls proliferation, differentiation, and other functions in many cell types		(11)

**Figure 1:**
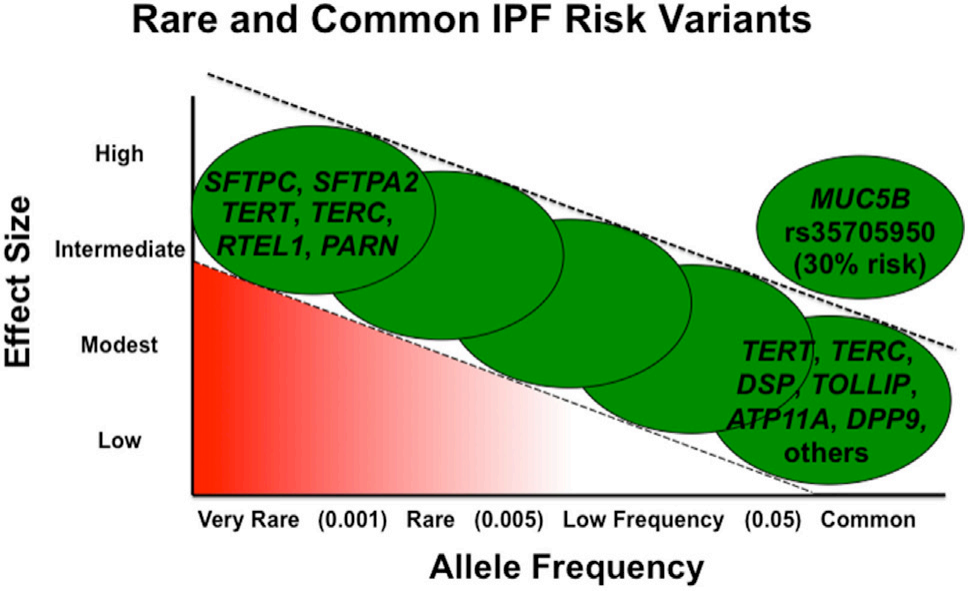
Adapted from figure published previously in BMC Medicine with permission (52).

Rare variants are thought to be highly penetrant and to have a greater effect size, but given their low frequency, they account for a smaller proportion of overall disease risk in the general population (51). Alternatively, in general, common variants have a smaller effect size but are present at higher frequency and, in aggregate, may contribute to a larger proportion of disease risk (Figure 1). However, the *MUC5B* promoter variant rs35705950 is a common variant with a large effect size and therefore accounts for a substantial risk in IPF. In fact, it has been estimated the *MUC5B* promoter variant accounts for 30% of the risk of developing IPF (41, 51).

### Alveolar Stability

Surfactant proteins are synthesized in the endoplasmic reticulum (ER) of alveolar type II cells (AECII) and transported to and stored in the lamellar bodies until secretion into the alveolar space (25, 26). Rare variants identified in the genes encoding surfactant protein C and A (*SFTPC, SFTPA1*, and *SFTPA2*) have been associated with pulmonary fibrosis (53). SP-C is a small hydrophobic protein produced by AECIIs that requires the C-terminus for initial folding steps in the ER before secretion into the alveolar space (26). *SFTPC* rare variants are mutations that lie in the BRICHOS domain within the C-terminus of SP-C. The BRICHOS domain is critical for proper folding and trafficking (5, 26). Coding mutations in this region lead to accumulation of misfolded protein resulting in increased ER stress and activation of the unfolded protein response (26, 54). Mutations in the gene that encodes surfactant protein A (*SFTPA2*) have also been linked to FIP (15) and have been associated with increased ER stress as well (28, 55). Rare variants have also been identified in another gene involved with surfactant processing, *ABCA3*, in FIP families (16, 56). *ABCA3* is a transporter protein mainly expressed in AECIIs and is involved in the transport of lipids across plasma membranes (29, 57). In AECIIs, *ABCA3* mutations cause abnormal processing, trafficking, and functionality of the *ABCA* protein, leading to retention of lipids in the ER, ER stress, and apoptotic signaling (30). These mutations are expressed in a recessive manner, where as mutations in *SFTPA2* and *SFTPC* are dominantly expressed (56).

In 2011, Lawson et al. (58) demonstrated that fibrotic remodeling in response to low-dose bleomycin was more severe in mice in which ER stress was induced, either through mutant SFTPC in AECIIs or by administration of tunicamycin, a chemical known to induce ER stress. In addition to effects on apoptosis, ER stress may induce biological pathways involved in cell differentiation (59, 60) through which epithelial cells acquire phenotypic characteristics of mesenchymal cells, a process known as epithelial-to-mesenchymal transition (EMT), in IPF lungs (61). EMT is hypothesized to increase the number of cells responsible for collagen production and matrix deposition thereby leading to fibrosis (13, 59, 60). To date, published data suggest that ER stress predisposes to AECII apoptosis and subsequent lung fibrosis. Surfactant proteins have been recognized as crucial in maintaining lung alveolar structure and function. However, the precise role of alveolar stability, ER stress, and EMT in IPF pathogenesis remains an area of active investigation.

### Cell Senescence

Telomeres are repetitive nucleotide sequences at the ends of chromosomes that protect them from progressive shortening during the normal cell replication process (62). Telomerases restore telomere length and consist of two major components: telomerase reverse transcriptase (encoded by *TERT*) and telomerase RNA (encoded by *TERC*) (18, 37). Mutations in telomerase components were initially identified in the setting of dyskeratosis congenita (DKC), a rare inherited syndrome of telomere shortening characterized by oral leukoplakia, abnormal skin hyperpigmentation, and dystrophic nails, with pulmonary fibrosis present in about 20% of patients; bone marrow failure can also be a complication of DKC (32). More recent studies have found an association between numerous genes in the telomerase maintenance pathway and FIP, including those related to catalytic activity (*TERT* and *TERC*) (7, 32) and telomere stabilization (*DKC1, PARN*, and *RTELI*) (19, 21). These pathogenic variants cause dysfunction of telomerase activity leading to accelerated telomere shortening (32, 63) in peripheral blood and the lung (32–34). Thus far, *TERT* variants are the most frequently identified rare variants associated with pulmonary fibrosis; they are found in ~15% of FIP (7, 32) and in 1–3% of sporadic cases (34). A recent whole-exome sequencing study identified *TERT, RTEL1*, and *PARN* variants previously associated with FIP to be associated with sporadic IPF, further supporting the role of telomere dysfunction in IPF pathogenesis and highlighting the genetic commonalities between FIP and sporadic IPF (64).

Telomere dysfunction has further been implicated in IPF as evidence has suggested that short telomeres are not exclusively related to telomerase rare variant mutations. One study found that 25% of sporadic IPF subjects and 24% of familial IPF subjects, without identified mutations for *TERT* or *TERC*, had short telomeres. In addition, all subjects within this specific study who had a mutation in *TERT* or *TERC* and pulmonary fibrosis also had short telomeres (33).

The mechanisms by which telomere defects provoke lung disease are not fully understood. Defects in telomere maintenance have been linked to epithelial cell senescence and an impaired response to epithelial injury (65). During successive cycles of cell division, telomere shortening occurs and eventually leads to activation of the DNA-damage pathways, which result in apoptosis or senescence (32). In certain situations, cellular senescence is appropriate, but premature senescence can impair lung epithelial homeostasis and lead to stimulation of a lung remodeling response (66), resulting in fibrotic lesions (63). One study demonstrated increased epithelial cell senescence in IPF lung tissue by measuring B-galactosidase staining (a marker of senescence) and found that B-galactosidase staining was positive in all IPF cases but was not present in normal lung (67) supporting a role for senescent epithelial cells in IPF pathogenesis. Future studies are necessary to clarify the precise role of cellular senescence in lung injury response and fibrotic remodeling in IPF.

### Host Defense

In 2011, genome-wide linkage analysis and targeted genetic sequencing identified a single nucleotide polymorphism (SNP) on chromosome 11 that was associated with both FIP and IPF (41). The SNP, rs35705950, was found to be a gain-of-function variant associated with increased expression of *MUC5B*. Heterozygous (GT) and homozygous (TT) individuals had an odds ratio for developing disease of 6.8 and 20.8 for FIP, and 9.0 and 21.8 for IPF, respectively, supporting the strength of the SNP’s association with development of both IPF and FIP (41). *MUC5B* encodes Mucin 5B, which is a major gel-forming mucin in mucus and expressed by airway epithelial cells (68, 69). The association of the *MUC5B* promoter polymorphism with IPF has been replicated and confirmed in nine independent cohorts (10, 11, 49, 50, 70–74), including in a 2013 GWAS (OR for T minor allele = 4.51; 95% CI = 3.91–5.21; *P* = 7.21 × 10^−95^) (10). Additional genotyping studies have noted that the *MUC5B* variant is associated with disease in a Mexican cohort of IPF patients, but not in Asian cohorts (75). Most recently, a study of select loci in various European cohorts, including Czech and Greek IPF patients, also confirmed the association between rs35705950 and IPF (76).

*MUC5B* expression in IPF is localized in the distal airway, respiratory bronchiole, honeycomb cyst (77), and the bronchiolar epithelium (78). Overexpression of *MUC5B* in these areas of the lung and especially in the honeycomb cysts, which are a histopathological finding in IPF (77), further supports the notion that *MUC5B* is important in the pathogenesis of IPF. Evans and colleagues (51) hypothesize that IPF is caused by recurrent injury/repair/regeneration at the bronchoalveolar junction secondary to overexpression of MUC5B, mucociliary dysfunction, retention of particles, ER stress, and disruption of normal reparative and regenerative mechanisms in the distal lung (51).

Interestingly, the *MUC5B* promoter polymorphism may be specific to IIP, since studies have illustrated that rs35705950 is not associated with increased risk of sarcoidosis and scleroderma-related ILD, two other fibrotic lung diseases (10, 50, 70). However, recent data have shown that *MUC5B* rs357057950 variant is associated with radiographic evidence of interstitial lung abnormalities (ILA) studied in the Framingham cohort (79–81). Increasing age and number of copies of *MUC5B* promoter polymorphism were associated with ILA progression, which has been linked to increased mortality (80, 81). In addition, there are some data to suggest that rs357057950 genotype may be associated with higher likelihood of radiographic UIP pattern in the setting of fibrotic IIP (82).

The mechanism by which variants in *MUC5B* confers risk of lung fibrosis is an active area of investigation. Given that mucins play a role in innate immunity (68, 83), immune dysregulation could be a possible mechanism by which increased mucin expression contributes to the pathophysiology of IPF (84). Alternatively, IPF may be a disease of mucociliary clearance in which overexpression of *MUC5B* leads to impaired ciliary function, thereby allowing retention of particles and, subsequently, recurrent lung injury (51).

Several studies have also implicated the human leukocyte antigen (HLA) region in IPF (85–89). The HLA region is located on chromosome 6p21.31 (90), and its main function is regulation of immune response. The DRB1*15:01 allele has been shown to be more prevalent among IPF patients and associated with greater impairment of gas exchange (89). Recently, a genome-wide imputation-based association analysis identified two risk alleles, DRB1*15:01 and DQB1*06:02, found to be associated with fibrotic idiopathic interstitial pneumonias (48). Although not definitive, HLA association with IPF may suggest that autoimmunity may play a role in pulmonary fibrosis; further characterizing the pathophysiologic connection between this genetic variation and disease this remains an area of active investigation.

### Epithelial Integrity

The 2013 GWAS by Fingerlin et al. (10) identified multiple susceptibility loci for fibrotic IIP, including two cell–cell adhesion molecules, *DSP* and *DPP9*. *DSP* gene expression was increased in lung tissue of individuals with IIP and varied by genotype for a variant in intron 5 (10, 91). *DSP* encodes for desmoplakin, a critical component of desmosome structure important in cell–cell adhesion. Desmosomes mechanically link cells and stabilize tissue architecture. In addition, they are involved in the regulation of cell proliferation, differentiation, migration, and apoptosis (92). The association between *DSP* variants and IPF, as well as the relationship between *DSP* variants and lung expression of this gene, was confirmed more recently by Mathai et al. (91) IPF lung has higher gene expression of *DSP*. However, IPF subjects with the rs2076295 variant were found to have lower *DSP* expression, suggesting that differential *DSP* expression may play a role in a subset or sub-phenotype of IPF (91). This association further implicates the airway epithelium in the pathogenesis of IPF, as *DSP* appears to be localized primarily to the airway epithelia and not to alveolar epithelial cells. The role of *DSP* in IPF pathogenesis remains an area of active investigation.

## Prognosis

Genetic variants, both rare (telomere related) and common (*MUC5B* and *TOLLIP*), may play a role in predicting disease outcomes and have prognostic implications. Short telomeres (<10th percentile adjusted for age) have been identified in a considerable portion of IPF patients, regardless of genetic mutations (33, 34). Patients with shorter telomeres have worse transplant-free survival in multiple independent cohorts (46, 93). Furthermore, a small observational study suggested that increased rates of bone marrow suppression and medication-related complications following lung transplantation are more common in IPF patients with telomerase mutations and/or short telomeres (94). Telomere length testing has been suggested as a component of pretransplant workup in IPF patients, although further prospective study is required before these observations can be utilized routinely in patient care (95).

Common polymorphisms, *MUC5B* and *TOLLIP*, have also shown promise as prognostic indicators (11, 50). A retrospective study of two separate IPF cohorts demonstrated improved survival in patients with the rs35705950 variant (49). In addition, carriers of at least 1 T allele of *MUC5B* polymorphism were found to have at least 50% improved survival and better lung function compared to those with the GG genotype (49). These findings were consistent with previous studies, which demonstrated an association between *MUC5B* variant and less severe pathological changes (96) and slower decline in FVC (50). Similarly, a *TOLLIP* variant was also associated with differential survival. The minor allele at rs5743890 (G) in TOLLIP is protective and associated with reduced susceptibility to IPF. However, those who developed IPF despite having the protective allele had increased mortality (11). At this time, there are no clinical guidelines suggesting genetic testing in the routine care and counseling of IPF patients (95), and further research is needed to identify the clinical implications of these preliminary findings.

## Treatment

Approaches to therapy in IPF have been limited by the poorly understood pathophysiology of this progressive disease. In addition, the unpredictable clinical course of IPF, lack of validated biomarkers, and low clinical predictive value to animal models (97) have been barriers to identifying therapies. Despite these challenges, recent advances in understanding the pathophysiology of IPF have allowed for identification of novel treatment targets. Currently, two available medications, pirfenidone (4) and ninetedanib (3), have been shown to reduce the rate of lung function decline among IPF patients. However, neither approved drug is curative.

With survival-associated variants (e.g., *MUC5B* and *TOLLIP*) (74), it is possible that genotypes will define subtypes with differential responses to therapy. Identifying distinct sub-phenotypes in IPF may enable the application of targeted therapy on a pathway-specific basis. For example, it may be possible to use telomere length or *TERT* genotype to identify a group of patients who would benefit from telomere-directed therapy (95). Oldham and colleagues (98) found that some carriers with *TOLLIP* polymorphism may benefit from treatment with oral *N*-acetylcysteine (NAC). More specifically, of those that received NAC, subjects with TT genotype for rs3750920 (*TOLLIP*) had decreased risk for the trial’s composite end point of death, hospitalization, or 10% decrement in forced vital capacity. In contrast, subjects with the CC genotype for rs3750920 had increased risk for the composite endpoints of the NAC intervention study (98). While NAC has not been shown to be effective in IPF in aggregate (99), it is possible that patients have differential response to this therapy (or other therapies) based on *TOLLIP* genotype (100). More prospectively designed studies are needed before genetic variation can be utilized routinely when choosing therapies for individual patients.

## Conclusion

This review focuses on the relationship between genetic variants and IPF. In addition to sequence variation, epigenetic changes (such as DNA methylation) (101–104) and gene expression changes are associated with disease risk and phenotype (103, 105, 106). Further studies are necessary to better understand the relationships between genetic variation and epigenetic and gene expression variation in terms of disease risk and phenotype.

Given the growing body of evidence that genetic variants influence disease risk as well as disease progression and clinically meaningful patient outcomes, it will be critical to account for genetic variation in future clinical trials. Such prospective studies and analyses that focus on the relationship between genotype and therapeutic response will be crucial in personalizing and improving IPF therapy.

## Author Contributions

AK and SM researched and wrote first draft of manuscript; DS edited and revised document. All the authors read and approved final version of manuscript.

## Conflict of Interest Statement

The authors declare that the research was conducted in the absence of any commercial or financial relationships that could be construed as a potential conflict of interest.
